# Supplementary material for: Tamsulosin use in benign prostatic hyperplasia and risks of Parkinson’s disease, Alzheimer’s disease and mortality: An observational cohort study of elderly Medicare enrollees
Source: PLoS One. 2024 Aug 22;19(8):e0309222. doi: 10.1371/journal.pone.0309222 (PMC11340942; doi:10.1371/journal.pone.0309222)
Supplement: Attachment — Submitted filename: Response to reviewers First revision.docx [file pone.0309222.s001.docx]

Dear PLOS ONE Editors,

As authors of the manuscript “Tamsulosin use for benign prostatic hyperplasia associated with increased risk of Parkinson Disease, Alzheimer Disease and mortality” we would like to thank the editors and reviewers for their thoughtful comments. We believe we have addressed all the concerns as detailed below.

**Comments to the Author**

1. Is the manuscript technically sound, and do the data support the conclusions?

The manuscript must describe a technically sound piece of scientific research with data that supports the conclusions. Experiments must have been conducted rigorously, with appropriate controls, replication, and sample sizes. The conclusions must be drawn appropriately based on the data presented.

Reviewer #1: Yes

2. Has the statistical analysis been performed appropriately and rigorously?

Reviewer #1: Yes

3. Have the authors made all data underlying the findings in their manuscript fully available?

The [PLOS Data policy](https://gcc02.safelinks.protection.outlook.com/?url=http%3A%2F%2Fwww.plosone.org%2Fstatic%2Fpolicies.action%23sharing&data=05%7C02%7Ckfung%40mail.nih.gov%7C71afd2346f8f40ddcf7508dca2b9083b%7C14b77578977342d58507251ca2dc2b06%7C0%7C0%7C638564163349308785%7CUnknown%7CTWFpbGZsb3d8eyJWIjoiMC4wLjAwMDAiLCJQIjoiV2luMzIiLCJBTiI6Ik1haWwiLCJXVCI6Mn0%3D%7C0%7C%7C%7C&sdata=Spro5vsiklLvs6e8Vyb%2FSHBu6Nsun58LU0jDi6Ys0%2Fc%3D&reserved=0) requires authors to make all data underlying the findings described in their manuscript fully available without restriction, with rare exception (please refer to the Data Availability Statement in the manuscript PDF file). The data should be provided as part of the manuscript or its supporting information, or deposited to a public repository. For example, in addition to summary statistics, the data points behind means, medians and variance measures should be available. If there are restrictions on publicly sharing data—e.g. participant privacy or use of data from a third party—those must be specified.

Reviewer #1: Yes

4. Is the manuscript presented in an intelligible fashion and written in standard English?

PLOS ONE does not copyedit accepted manuscripts, so the language in submitted articles must be clear, correct, and unambiguous. Any typographical or grammatical errors should be corrected at revision, so please note any specific errors here.

Reviewer #1: No

>> the manuscript has been revised by professional copyeditors to improve the readability and quality of writing

5. Review Comments to the Author

Please use the space provided to explain your answers to the questions above. You may also include additional comments for the author, including concerns about dual publication, research ethics, or publication ethics. (Please upload your review as an attachment if it exceeds 20,000 characters)

Reviewer #1: I would like to congratulate the authors for the novel study that explores the risk of PD among AB drug users. However, I 've noticed a few issues within the manuscript which are listed as follows:

1. The flow and language of the manuscript is fair. But I believe the language and grammar can be brushed up further to improve the quality.

>> the manuscript has been revised by professional copyeditors to improve the readability and quality of writing

2. This manuscript misses to discuss a recently published meta-analysis on the same question: Lamichhane P, Tariq A, Akhtar AN, Raza M, Lamsal AB, Agrawal A. Risk of Parkinson's disease among users of alpha-adrenergic receptor antagonists: a systematic review and meta-analysis. Ann Med Surg (Lond). 2024 May 6;86(6):3409-3415. doi: 10.1097/MS9.0000000000002117. PMID: 38846867; PMCID: PMC11152853.

>> Thanks for the suggestion. The said reference, together with 2 other relevant references have been added and discussed.
3. Please add statement regarding ethical approval from ethical review committee and need of consent from the study subjects.

>> This has been included in the manuscript: “This study was declared not human subject research by the Office of Human Research Protection at the National Institutes of Health and by the CMS’s Privacy Board.” The documentation of the determination has been updated separately through the submission website.
